# Prevalence and Risk of Infection in Patients with Diabetes following Primary Total Knee Arthroplasty: A Global Systematic Review and Meta-Analysis of 120,754 Knees

**DOI:** 10.3390/jcm11133752

**Published:** 2022-06-28

**Authors:** Mohd Aliff Ahmad, Shaifuzain Ab Rahman, Md Asiful Islam

**Affiliations:** 1 Department of Orthopaedics, School of Medical Sciences, Universiti Sains Malaysia, Kubang Kerian 16150, Kelantan, Malaysia; drmohdaliff@gmail.com; 2 Department of Haematology, School of Medical Sciences, Universiti Sains Malaysia, Kubang Kerian 16150, Kelantan, Malaysia; 3 Institute of Metabolism and Systems Research, University of Birmingham, Birmingham B15 2TT, UK

**Keywords:** diabetes, infection, periprosthetic joint infection, prevalence, risk, systematic review, total knee arthroplasty

## Abstract

Diabetes mellitus (DM) is a known risk factor for infection following total joint arthroplasty. This study looked at the prevalence and risk of infection in diabetic and non-diabetic patients who had primary total knee arthroplasty (TKA). PubMed, Scopus, Google Scholar, Web of Science, and Science Direct electronic databases were searched for studies published up to 21 April 2022. To compare the risk of infection between diabetic and non-diabetic subjects, a pooled prevalence, and a risk ratio (RR) with 95% confidence intervals (CIs) were used. This research has been registered with PROSPERO (CRD42021244391). There were 119,244 participants from 18 studies, with a total of 120,754 knees (25,798 diabetic and 94,956 non-diabetic). We discovered that the risks of infection in diabetic patients were 1.84 times significantly higher than in non-diabetic patients. Infection was more common in diabetic patients (1.9%) than in non-diabetic patients (1.2%). In a subgroup analysis, the risks of developing deep surgical site infection (SSI) were 1.96 times higher in diabetic patients, but no significant difference when compared in superficial SSI. Prevalence of deep SSI was higher in diabetic (1.5%) than in non-diabetic (0.7%), but the prevalence of superficial SSI was lower in diabetic (1.4%) than in non-diabetic (2.1%). Consistent with previous research, we found diabetes is a risk factor for infection following primary TKA. However, the risk is much lower than previously published data, indicating that other factors play a larger role in infection.

## 1. Introduction

Total knee arthroplasty (TKA) has long been considered the most effective surgery for patients suffering from severe knee arthritis [1]. It is a major operation that is frequently used to relieve joint pain and improve joint mobility and function [2]. Though it is uncommon, postoperative infection is one of the most devastating and feared complications of TKA [3,4].

Diabetes mellitus (DM) is one of several risk factors for periprosthetic joint infection (PJI) following total joint arthroplasty [5]. It has been reported that more than half of those with diabetes have arthritis and may require a hip or knee replacement in the future [6,7]. With the increasing prevalence of diabetes worldwide, the number of diabetic patients requiring arthroplasty is expected to rise in the future [8]. Therefore, we believe that studies on risk infection in diabetics undergoing TKA will be extremely beneficial in preventing PJI.

To the best of our knowledge, no recent systematic review and meta-analysis has been conducted comprehensively to investigate the prevalence and risk of infection in patients with diabetes following primary TKA, with the most recent known being in 2014 which was on the influence of DM on the post-operative outcome of elective primary TKA. The objective of this meta-analysis was to provide a recent update on the comparison of infection prevalence and risk in diabetic and non-diabetic patients undergoing primary TKA. This study aims to assist surgeons to understand the current scenario and in improving treatment strategies for diabetic patients undergoing TKA.

## 2. Methods

In accordance with the Preferred Reporting Items for Systematic Reviews and Meta-analyses (PRISMA) guideline, we conducted this systematic review and meta-analysis to assess the prevalence and risk of infection among diabetic patients compared to non-diabetic subjects who receive primary TKA [9]. The protocol of this study was registered with International Prospective Register of Systematic Reviews (PROSPERO) database, registration number: CRD42021244391.

### 2.1. Data Sources and Searches

PubMed, Scopus, Google Scholar, Web of Science, and Science Direct electronic databases were searched and identified studies published from inception to 21 April 2022. We looked over the reference lists of the included studies for other potential studies that could be included in the SRMA. EndNote X8 software was used to manage and screen out duplicate studies.

### 2.2. Eligibility Criteria

We considered observational studies as eligible studies. Preprints were not considered and only published studies reporting data of interest were considered eligible. Review papers, case studies, comments, and perspectives were not included in the study. Data from news reports and press releases, as well as data gathered from websites and databases, were not taken into account. Studies published in languages other than English were included, with Google Translate used to translate them. We were cautious about studies from the same authors or facilities, but if the study population was distinct, the study was included.

### 2.3. Inclusion and Exclusion Criteria

Patients with diabetes who had a primary TKA were included in the study and compared with non-diabetic subjects. Research involving (1) revision total knee replacement, (2) original knee replacement with evidence of prior infection, and (3) animal studies are excluded. At the same time, we also excluded data obtained from insurance companies and from hospital billing.

### 2.4. Study Selection

Articles of interest were reviewed based on title and abstract, then full text by two authors (M.A.A. and S.A.R.) separately to find suitable studies. Disagreements over inclusion were aired and a consensus was reached by discussing among the authors.

### 2.5. Data Extraction

Data extraction was done by M.A.A. and cross-checked independently by two authors (M.A.I. and S.A.R.). When duplicate data were discovered, the study with the smaller sample size or incomplete data was discarded. We took the following data from each eligible study and entered it into a pre-set Excel spreadsheet: the first author’s last name; the participants’ region (country); the data collecting period; the total number of TKA patients; the total number of knees examined; age; type of infection and the study design.

### 2.6. Quality Assessment

The quality of included studies was assessed independently by two authors (M.A.A. and S.A.R.) using the Joanna Briggs Institute (JBI) critical appraisal tools [10]. Further, the results of the quality assessment were checked by another author (M.A.I.). Studies were categorised as “high risk of bias” (low quality), “moderate risk of bias” (moderate quality) or “low risk of bias” (high quality) when the overall score was <50%, 50–70% or >70%, respectively [11,12].

### 2.7. Data Analyses

The pooled prevalence and 95% confidence intervals (CIs) of infection in diabetes patients were calculated using a random-effects model. The risk ratio (RR) with 95% confidence intervals (CIs) was used to compare the risk of infection between diabetic and non-diabetic subjects. In addition, the pooled prevalence of infection with the corresponding 95% CI were calculated for both diabetic and non-diabetic subjects.

To examine publication bias, funnel plots displaying prevalence estimates versus sample variance were created, and the asymmetry of the funnel plot was confirmed using Egger’s test when a minimum of 10 studies were available. Heterogeneity between studies was assessed using the *I*^2^ statistic (*I*^2^ > 75% indicating substantial heterogeneity) in addition to using Cochran’s Q test to identify the significance of heterogeneity. Galbraith plots were constructed to identify the sources of heterogeneity. Subgroup analysis was done by analysing the risk and prevalence of deep surgical site infection and superficial infection. Sensitivity analyses were performed by (A) leave-one-out method, (B) excluding the outlier studies, (C) excluding small studies (*n* < 500 for RR estimation and *n* < 100 for prevalence estimation) and (D) excluding low- and moderate-quality studies. All the analyses and plots were generated by using metaprop codes in meta (version 4.11–0) and metafor (version 2.4–0) packages of R (version 3.6.3) in RStudio (version 1.2.5033) and RevMan (version 5.3) software [13,14].

## 3. Results

### 3.1. Study Selection

**Figure 1:**
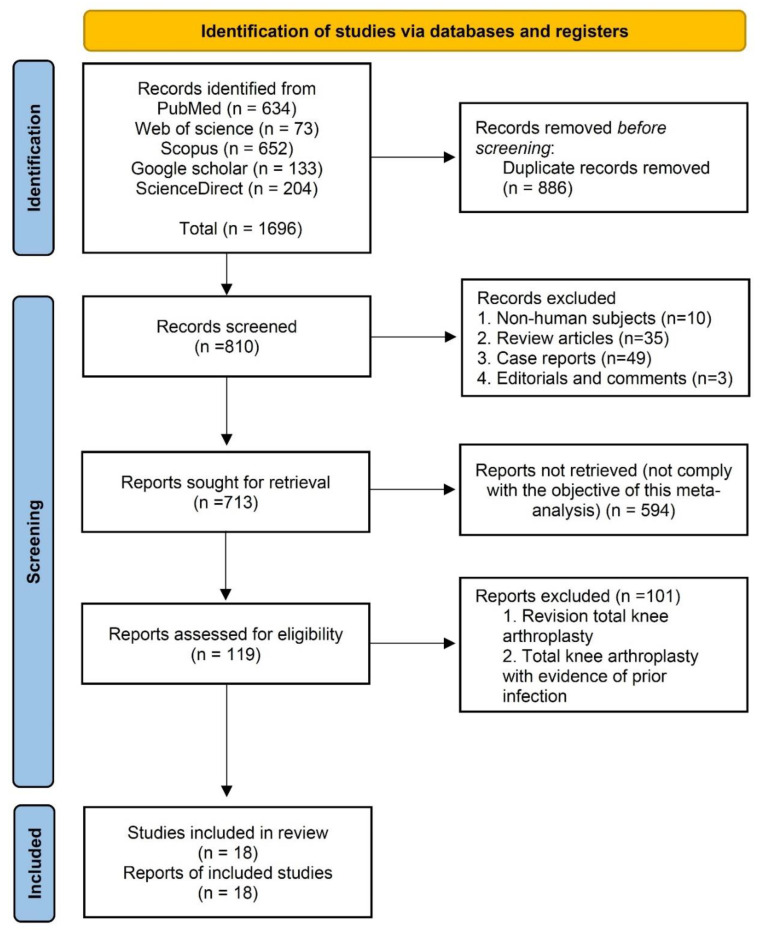
PRISMA flow diagram of study selection.

Initially, 1696 articles were identified from the five databases. Following the removal of 983 articles (based on the following criteria: non-human subject, review article, case reports, editorial, comments, and duplicate studies), another 594 studies were excluded from the remaining articles based on title and/or abstract evaluation. Furthermore, 101 articles were excluded with reasons during the full text review. Finally, 18 of the articles met the eligibility criteria and were included in the systematic review and meta-analysis (Figure 1).

### 3.2. Characteristics of Included Studies

This meta-analysis is based on a study of 119,244 participants, with total of 120,754 knees (25,798 diabetic and 94,956 non-diabetic) included. The studies were conducted in 10 different countries: the USA (*n* = 6), the UK (*n* = 1), Taiwan (*n* = 1), Spain (*n* = 2), South Korea (*n* = 2), Singapore (*n* = 1), Pakistan (*n* = 1), Japan (*n* = 1), India (*n* = 1) and Hong Kong (*n* = 2). A total of 14 studies were case-control studies while the remaining 4 were cohort studies. All the papers included data of deep surgical site infection (SSI). Out of these 18 studies, 9 papers also included superficial SSI in their analysis. The detailed characteristics of the included studies are summarised in Table 1.

### 3.3. Primary Outcomes

**Figure 2:**
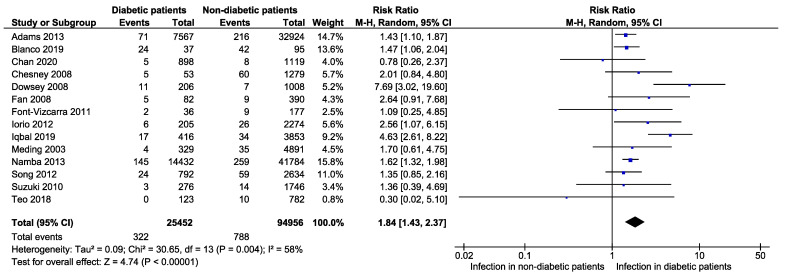
Risk of developing an infection in diabetic patients compared to non-diabetic patients following primary total knee arthroplasty [1,15,16,17,18,20,21,22,24,25,26,27,30,31].

**Figure 3:**
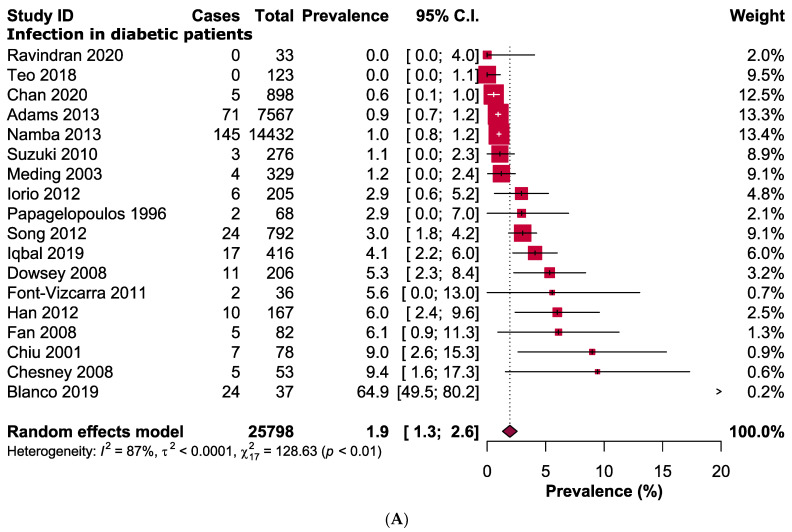

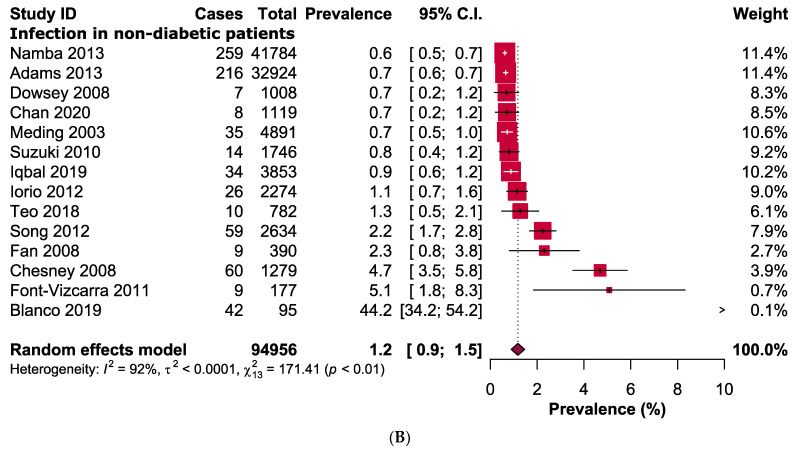
Prevalence of infection in (**A**) diabetic and (**B**) non-diabetic patients following primary total knee arthroplasty [1,15,16,17,18,19,20,21,22,23,24,25,26,27,28,29,30,31].

From our analysis, we found that risks of developing infection in diabetic patients were 1.84 times higher than that in non-diabetic patients following a primary TKA (RR: 1.84, 95% CI: 1.43–2.37, *p* < 0.00001, *I*^2^ = 58%, *n* = 25,452 vs. 94,956) (Figure 2). It also established that prevalence of infection was higher in diabetic patients (1.9%, 95% CI: 1.3–2.5%; *I*^2^ = 87%, *n* = 25,798) in compared to non-diabetic patients (1.2%, 95% CI: 0.9–1.5%, *I*^2^ = 92%, *n* = 94,956) following TKA (Figure 3).

**Figure 4:**
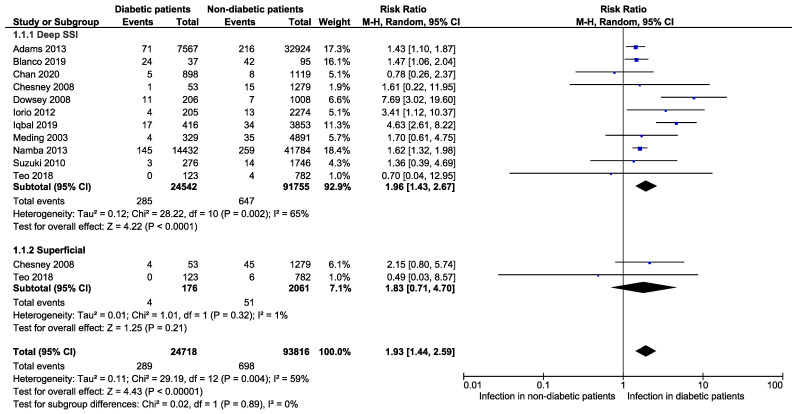
Subgroup analysis representing the risk of developing deep surgical site infection and superficial infection in diabetic patients compared to non-diabetic patients following primary total knee arthroplasty [1,15,16,17,18,24,25,26,27,31].

**Figure 5:**
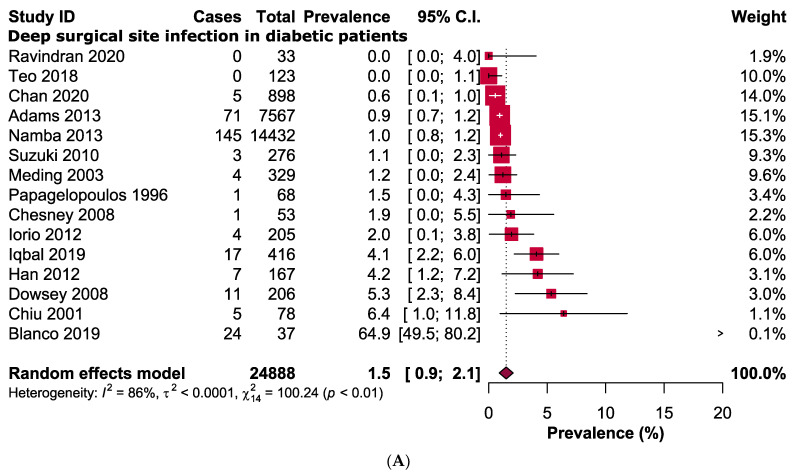

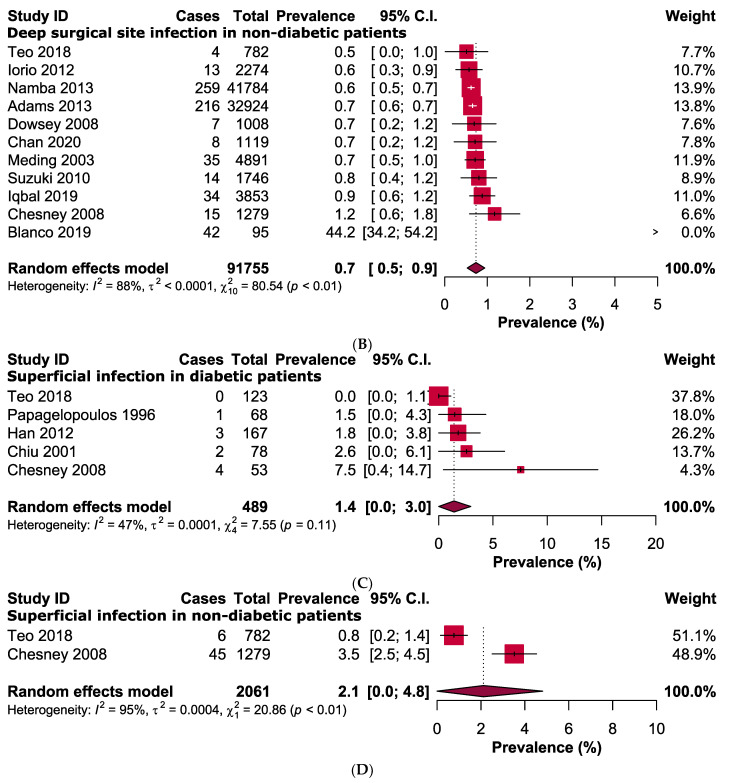
Prevalence of deep surgical site infection in (**A**) diabetic and (**B**) non-diabetic patients and superficial infection in (**C**) diabetic and (**D**) non-diabetic patients following primary total knee arthroplasty [1,15,16,17,18,19,20,23,24,25,26,27,28,29,31].

Further subgroup analysis for comparison between superficial and deep SSI was carried out. Risks of developing deep SSI in diabetic patients were 1.96 times higher than that in non-diabetic patients following a primary TKA (RR: 1.96, 95% CI: 1.43–2.67, *p* < 0.0001, *I*^2^ = 65%, *n* = 25,452 vs. 91,755) (Figure 4), however no significant difference when comparing it in the superficial SSI group (RR: 1.83, 95% CI: 0.71–4.70, *p* = 0.21, *I*^2^ = 1%, *n* = 176 vs. 2061) (Figure 4). Prevalence of deep SSI was higher in diabetic patients (1.5%, 95% CI: 0.9–2.1%; *I*^2^ = 86%, *n* = 24,888) in compared to non-diabetic patients (0.7%, 95% CI: 0.9–0.9%, *I*^2^ = 88%, *n* = 91,755) following TKA (Figure 5). However, the prevalence of superficial SSI was lower in diabetic patients (1.4%, 95% CI: 0.0–3.0%; *I*^2^ = 47%, *n* = 489) in compared to non-diabetic patients (2.1%, 95% CI: 0.0–4.8%, *I*^2^ = 95%, *n* = 2061).

### 3.4. Quality Assessment and Publication Bias

**Figure 6:**
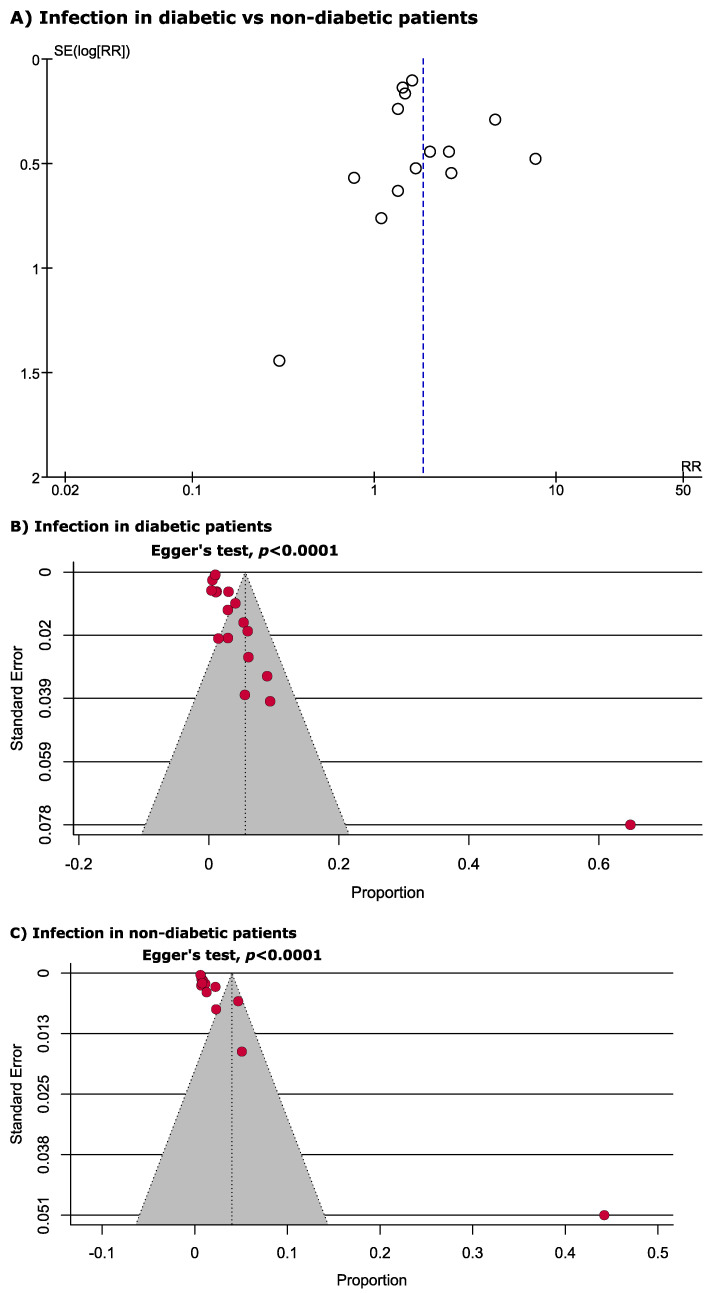
Funnel plots identified significant publication bias estimating the (**A**) risk ratio and prevalence of infection in (**B**) diabetic and (**C**) non-diabetic patients following primary total knee arthroplasty.

**Figure 7:**
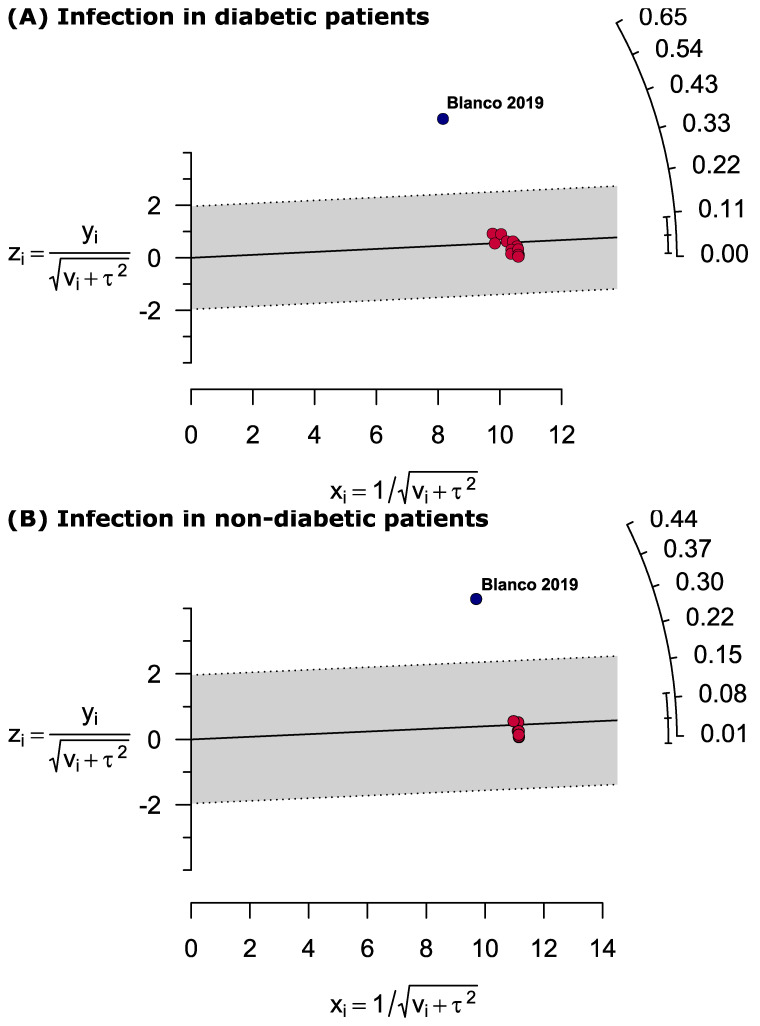
Galbraith plots identified Blanco 2019 as the potential source of heterogeneity estimating the prevalence of infection in (**A**) diabetic and (**B**) non-diabetic patients following primary total knee arthroplasty.

Table S3 shows the quality assessment of all 18 studies. Based on our assessment, 88.8% (*n* = 16) of the included studies were of high quality (low risk of bias) while the rest, 11.1% (*n* = 2), were of moderate quality (moderate risk of bias). None of the included studies were classified as low quality (high risk of bias). We observed significant publication bias estimating both risk ratio and prevalence of infection in diabetic and non-diabetic patients following primary TKA (Figure 6). The Galbraith plot identified Blanco 2019 as the outlier study estimating the prevalence of infection in both diabetic and non-diabetic patients. From RR estimation, using the leave-one-out method, we identified two outlier studies (Iqbal 2019 and Dowsey 2008) (Figure 7) and, interestingly, after excluding these two outlier studies from the analysis, the heterogeneity becomes zero.

### 3.5. Sensitivity Analyses

From the sensitivity analyses (Table 2), it was evident that there was no remarkable difference in results after we excluded outlier studies, small studies, low- and moderate-quality studies, indicating that our main results estimating both RR and prevalence of infection in diabetic and non-diabetic patients following primary TKA was robust and reliable.

## 4. Discussion

According to our meta-analysis, the prevalence of infection after primary TKA was higher in diabetes patients (1.9%) than in non-diabetic patients (1.2%). This is not surprising given that diabetes is a known independent risk factor for PJI [32]. Long-term hyperglycaemia had a deleterious effect on the immune system due to reduced leukocyte activity, which raised the risk of perioperative superficial and deep tissue infections, according to previous study on the interaction between DM and the immune system [33]. Furthermore, diabetes mellitus may impede wound healing because microangiopathic alterations may lower antibiotic concentrations in the tissue, resulting in local tissue ischaemia [34].

Even though it is generally recognised that diabetes is a known risk factor for SSI, it is intriguing that we discovered that the chance of diabetics developing SSI is only 1.84 times, compared to 3.72 times in a prior meta-analysis published in 2013. This conclusion is based on newly available data from research conducted after 2013 [17,31]. Several factors influenced this outcome, including the length of the operation [31] and pre-operative glycaemic control optimization [17]. Controlling hyperglycaemia in the perioperative period can minimise the likelihood of infection in patients receiving THA and TKA, according to an observational study by Agos et al. [35]. As a result, we advocate optimum glycaemic control and a shorter operative time as factors to consider when reducing the risk of infection in diabetics.

PJI is one of the most feared complications and is the most important cause of TKA revision [36]. Diabetic individuals had higher risks and prevalence of deep SSI (PJI) than non-diabetic patients, according to our findings. This finding is consistent with results published by Qin et colleagues in 2020, who found that patients with DM had a 1.76-fold greater risk of deep infection [37]. We believed that proper precautions should be taken when operating on diabetic patients for TKA, as previous research has shown that PJI is associated with extremely poor postoperative outcomes and a high mortality rate [22]. It is critical because as managing PJI is extremely expensive, putting a significant financial strain on the health-care system [38,39].

Even though the prevalence of superficial SSI in diabetics is lower than in non-diabetics, this could be due to a lack of data, as only two studies were suitable for this comparison (compared to diabetics—five). Most studies focus on PJI rather than superficial SSI because superficial SSI is easily treated with antibiotics and regular wound review [31]. We do recommend that future research should concentrate on superficial SSI in the study of primary TKA because it will provide a better understanding of the problem.

This research has numerous major advantages. To our knowledge, no meta-analysis has previously looked into the prevalence of infection after primary TKA in diabetics and non-diabetics. This meta-analysis used robust search algorithms with no language constraints to pull studies from five databases. All of the sensitivity analyses yielded similar results to the overall findings, implying that the primary discovery is likely to be reliable. This review, however, has its own limitations. Our ability to examine the associated risk is hampered by a lack of data, particularly on superficial SSI. Furthermore, the under reported level of glycaemic control in each paper limits our capacity to assess the impact of this factor on infection risk.

## 5. Conclusions

In conclusion, this SRMA confirms that diabetes is a risk factor for infection following primary TKA, which is consistent with previous research. However, the risk has been overemphasised and is not as severe as previously reported. This suggests that other factors, such as glycaemic control and operation duration, may have an impact on infection than diabetes in patients underwent primary TKA. There is insufficient evidence to conclude that diabetes is a risk factor for superficial SSI, in particular; therefore, more research is required to establish the relationship between DM and superficial SSI.

## Figures and Tables

**Table 1:** Major characteristics of the included studies.

Study ID	Country	Data Collection Period	Total Number of Knees (Diabetic/Non-Diabetics)	Total Number of Study Participants (Diabetic/Non-Diabetics)	Age of the Patients (Years) [Mean ± SD/Median (IQR)/Range]	Type of Infection	Study Design
Adams 2013 [15]	USA	2001–2009	40,491 (7567/32,924)	40,491 (7567/32,924)	61–75	Deep SSI	Case-control, retrospective
Blanco 2019 [16]	Spain	NR	132 (37/95)	132 (37/95)	Infected: 72.4 ± 7.0, Not infected: 70.1 ± 7.7	Deep SSI	Case-control, retrospective
Chan 2020 [17]	Hong Kong	December 2014–May 2019	2017 (898/1119)	1566 (704/862)	23.0–93.0	Deep SSI	Case-control, prospective
Chesney 2008 [18]	UK	October 1998–February 2005	1332 (53/1279)	1332 (53/1279)	64.0–75.0	Superficial and deep SSI	Case-control, prospective
Chiu 2001 [19]	Taiwan	1993–1998	78 (78/0)	78 (78/0)	69.0–72.0	Superficial and deep SSI	Cohort, prospective
Dowsey 2008 [20]	USA	1998–2005	1214 (206/1008)	1214 (206/1008)	72.0 (65.0–77.0)	Deep SSI	Case-control, retrospective
Fan 2008 [21]	Hong Kong	July 1997–June 2006	472 (82/390)	348 (NR/NR)	69.0 (40.0–88.0)	Superficial and deep SSI	Case-control, retrospective
Font-Vizcarra 2011 [22]	Spain	December 2007–May 2008	213 (36/177)	213 (36/177)	Infected: 77.0 (74.0–80.0), Not infected: 72.0 (66.0–78.0)	Superficial and deep SSI	Case-control, prospective
Han 2012 [23]	South Korea	January 2001–March 2007	167 (167/0)	115 (115/0)	68.0 (49.0–82.0)	Superficial and deep SSI	Cohort, retrospective
Iorio 2012 [24]	USA	December 2004–December 2009	2479 (205/2274)	2479 (205/2274)	NR	Superficial and deep SSI	Case-control, retrospective
Iqbal 2019 [25]	Pakistan	June 2008–December 2018	4269 (416/3853)	4269 (416/3853)	61.4 ± 10.2	Deep SSI	Case-control, retrospective
Meding 2003 [26]	USA	June 1987–November 1999	5220 (329/4891)	5220 (329/4891)	NR	Deep SSI	Case-control, prospective
Namba 2013 [27]	USA	April 2001–December 2009	56,216 (14,432/41,784)	56,216 (14,432/41,784)	67.4 ± 9.6	Deep SSI	Case-control, prospective
Papagelopoulos 1996 [28]	USA	May 1978–May 1982	68 (68/0)	51 (51/0)	NR	Superficial and deep SSI	Cohort, prospective
Ravindran 2020 [29]	India	July 2018–October 2019	33 (33/0)	33 (33/0)	48.0–71.0	Deep SSI	Cohort, prospective
Song 2012 [30]	South Korea	2006–2009	3426 (792/2634)	3426 (792/2634)	SSI: 67.0 ± 8.8, No SSI: 68.6 ± 7.5	Superficial and deep SSI	Case-control, retrospective
Suzuki 2010 [1]	Japan	1995–2006	2022 (276/1746)	1146 (NR/NR)	Infected: 69.5 ± 7.1, Not infected 70.7 ± 8.5	Deep SSI	Case-control, retrospective
Teo 2018 [31]	Singapore	February 2004–July 2014	905 (123/782)	905 (123/782)	65.9 ± 7.7	Superficial and deep SSI	Case-control, prospective

NR—not reported; SSI—surgical site infection.

**Table 2:** Sensitivity analyses.

Risk Ratio Estimation						
Strategies of Sensitivity Analyses	RR [95% CI]	Difference of Pooled RR Compared to the Main Result	Number of Studies Analysed	Total Number of Participants	Heterogeneity	
					*I^2^*	*p*-Value
Excluding outlier studies	1.5 [1.3–1.7]	0.31 lower	12	114,925	0%	0.83
Excluding small studies (*n* < 500)	1.9 [1.4–2.6]	0.09 higher	11	119,591	66%	0.001
Excluding low- and moderate-quality studies	1.8 [1.3–2.3]	0.003 lower	13	117,929	60%	0.003
**Prevalence estimation (diabetic patients)**						
**Strategies of sensitivity analyses**	**Prevalence** **[95% CI] %**	**Difference of pooled prevalence compared to the main result**	**Number of studies analysed**	**Total number of participants**	**Heterogeneity**	
					***I^2^***	***p*** **-value**
Excluding outlier studies	1.5 [1.1–2.0]	0.4 lower	17	25,761	74%	<0.0001
Excluding small studies (*n* < 100)	1.4 [0.9–1.8]	0.5 lower	11	25,411	78%	<0.0001
Excluding low- and moderate-quality studies	1.9 [1.3–2.6]	No difference	16	25,560	88%	<0.0001
**Prevalence estimation (non-diabetic patients)**						
Excluding outlier studies	1.1 [0.8–1.3]	0.1 lower	13	94,861	88%	<0.0001
Excluding small studies (*n* < 100)	1.2 [0.9–1.5]	No difference	14	94,956	92%	<0.0001
Excluding low- and moderate-quality studies	1.2 [0.9–1.5]	No difference	13	92,682	93%	<0.0001

RR—risk ratio, CIs—confidence intervals.

## Data Availability

Data is contained within the article or supplementary material.

## Supplementary Materials

The following supporting information can be downloaded at: www.mdpi.com/article/10.3390/jcm11133752/s1, Figure S1: Sensitivity analyses; Table S1: Search strategies; Table S2: Quality assessment of the included case-control studies; Table S3: Quality assessment of the included cohort studies.
