# Supplementary material for: Prevalence and Risk of Infection in Patients with Diabetes following Primary Total Knee Arthroplasty: A Global Systematic Review and Meta-Analysis of 120,754 Knees
Source: J Clin Med. 2022 Jun 28;11(13):3752. doi: 10.3390/jcm11133752 (PMC9267175; doi:10.3390/jcm11133752)
Supplement: Supplementary file 1 [file jcm-11-03752-s001.zip › jcm-1776430-supplementary.pdf]

A
Excluding outlier studies

Study or Subgroup	Diabetic patients Events	Diabetic patients Total	Non-diabetic patients Events	Non-diabetic patients Total	Weight	Risk Ratio M-H, Random, 95% CI
Adams 2013	71	7567	216	32924	23.9%	1.43 [1.10, 1.87]
Blanco 2019	24	37	42	95	15.9%	1.47 [1.06, 2.04]
Chan 2020	5	898	8	1119	1.4%	0.78 [0.26, 2.37]
Chesney 2008	5	53	60	1279	2.2%	2.01 [0.84, 4.80]
Dowsey 2008	11	206	7	1008	0.0%	7.69 [3.02, 19.60]
Fan 2008	5	82	9	390	1.5%	2.64 [0.91, 7.68]
Font-Vizcarra 2011	2	36	9	177	0.8%	1.09 [0.25, 4.85]
Iorio 2012	6	205	26	2274	2.2%	2.56 [1.07, 6.15]
Iqbal 2019	17	416	34	3853	0.0%	4.63 [2.61, 8.22]
Meding 2003	4	329	35	4891	1.6%	1.70 [0.61, 4.75]
Namba 2013	145	14432	259	41784	41.5%	1.62 [1.32, 1.98]
Song 2012	24	792	59	2634	7.8%	1.35 [0.85, 2.16]
Suzuki 2010	3	276	14	1746	1.1%	1.36 [0.39, 4.69]
Teo 2018	0	123	10	782	0.2%	0.30 [0.02, 5.10]
**Total (95% CI)**		**24830**		**90095**	**100.0%**	**1.53 [1.35, 1.75]**
Total events	294		747			

Heterogeneity: Tau² = 0.00; Chi² = 6.56, df = 11 (P = 0.83); I² = 0%

Test for overall effect: Z = 6.43 (P < 0.00001)

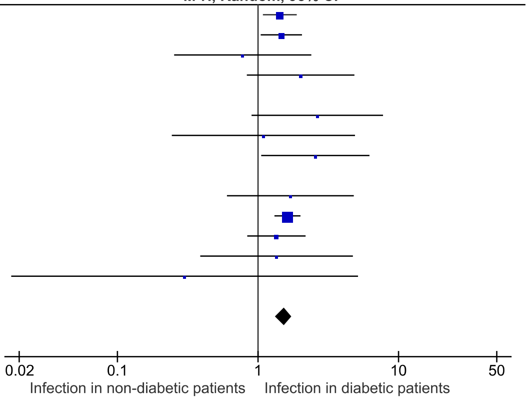

B
Excluding small studies

Study or Subgroup	Diabetic patients Events	Diabetic patients Total	Non-diabetic patients Events	Non-diabetic patients Total	Weight	Risk Ratio M-H, Random, 95% CI
Adams 2013	71	7567	216	32924	16.7%	1.43 [1.10, 1.87]
Blanco 2019	24	37	42	95	0.0%	1.47 [1.06, 2.04]
Chan 2020	5	898	8	1119	5.6%	0.78 [0.26, 2.37]
Chesney 2008	5	53	60	1279	7.8%	2.01 [0.84, 4.80]
Dowsey 2008	11	206	7	1008	7.1%	7.69 [3.02, 19.60]
Fan 2008	5	82	9	390	0.0%	2.64 [0.91, 7.68]
Font-Vizcarra 2011	2	36	9	177	0.0%	1.09 [0.25, 4.85]
Iorio 2012	6	205	26	2274	7.7%	2.56 [1.07, 6.15]
Iqbal 2019	17	416	34	3853	11.7%	4.63 [2.61, 8.22]
Meding 2003	4	329	35	4891	6.3%	1.70 [0.61, 4.75]
Namba 2013	145	14432	259	41784	17.6%	1.62 [1.32, 1.98]
Song 2012	24	792	59	2634	13.4%	1.35 [0.85, 2.16]
Suzuki 2010	3	276	14	1746	4.8%	1.36 [0.39, 4.69]
Teo 2018	0	123	10	782	1.2%	0.30 [0.02, 5.10]
**Total (95% CI)**		**25297**		**94294**	**100.0%**	**1.93 [1.41, 2.64]**
Total events	291		728			

Heterogeneity: Tau² = 0.14; Chi² = 29.09, df = 10 (P = 0.001); I² = 66%

Test for overall effect: Z = 4.08 (P < 0.0001)

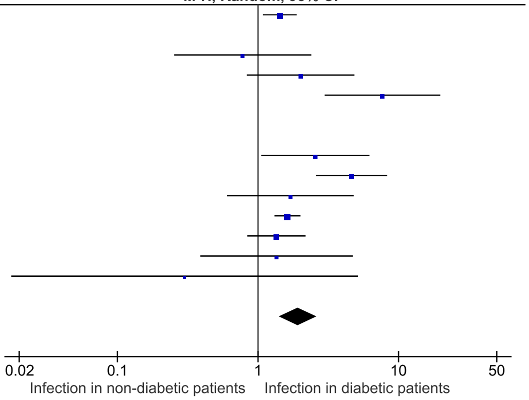

C
Excluding low- and moderate-quality studies

Study or Subgroup	Diabetic patients Events	Diabetic patients Total	Non-diabetic patients Events	Non-diabetic patients Total	Weight	Risk Ratio M-H, Random, 95% CI
Adams 2013	71	7567	216	32924	15.4%	1.43 [1.10, 1.87]
Blanco 2019	24	37	42	95	14.3%	1.47 [1.06, 2.04]
Chan 2020	5	898	8	1119	4.3%	0.78 [0.26, 2.37]
Chesney 2008	5	53	60	1279	6.1%	2.01 [0.84, 4.80]
Dowsey 2008	11	206	7	1008	5.5%	7.69 [3.02, 19.60]
Fan 2008	5	82	9	390	4.6%	2.64 [0.91, 7.68]
Font-Vizcarra 2011	2	36	9	177	2.7%	1.09 [0.25, 4.85]
Iorio 2012	6	205	26	2274	0.0%	2.56 [1.07, 6.15]
Iqbal 2019	17	416	34	3853	9.8%	4.63 [2.61, 8.22]
Meding 2003	4	329	35	4891	4.8%	1.70 [0.61, 4.75]
Namba 2013	145	14432	259	41784	16.5%	1.62 [1.32, 1.98]
Song 2012	24	792	59	2634	11.6%	1.35 [0.85, 2.16]
Suzuki 2010	3	276	14	1746	3.6%	1.36 [0.39, 4.69]
Teo 2018	0	123	10	782	0.8%	0.30 [0.02, 5.10]
**Total (95% CI)**		**25247**		**92682**	**100.0%**	**1.81 [1.39, 2.35]**
Total events	316		762			

Heterogeneity: Tau² = 0.10; Chi² = 29.69, df = 12 (P = 0.003); I² = 60%

Test for overall effect: Z = 4.42 (P < 0.0001)

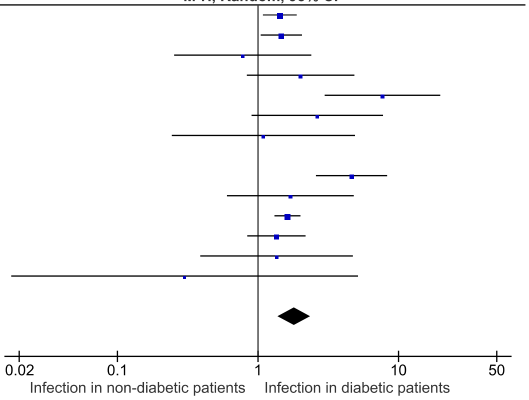

D

Study ID	Cases	Total	Prevalence	95% C.I.	Weight
**Diabetic: Excluding outlier studies**					
Ravindran 2020	0	33	0.0	[0.0; 4.0]	1.2%
Teo 2018	0	123	0.0	[0.0; 1.1]	8.9%
Chan 2020	5	898	0.6	[0.1; 1.0]	15.2%
Adams 2013	71	7567	0.9	[0.7; 1.2]	17.5%
Namba 2013	145	14432	1.0	[0.8; 1.2]	17.7%
Suzuki 2010	3	276	1.1	[0.0; 2.3]	8.1%
Meding 2003	4	329	1.2	[0.0; 2.4]	8.4%
Iorio 2012	6	205	2.9	[0.6; 5.2]	3.3%
Papagelopoulos 1996	2	68	2.9	[0.0; 7.0]	1.3%
Song 2012	24	792	3.0	[1.8; 4.2]	8.3%
Iqbal 2019	17	416	4.1	[2.2; 6.0]	4.5%
Dowsey 2008	11	206	5.3	[2.3; 8.4]	2.1%
Font-Vizcarra 2011	2	36	5.6	[0.0; 13.0]	0.4%
Han 2012	10	167	6.0	[2.4; 9.6]	1.5%
Fan 2008	5	82	6.1	[0.9; 11.3]	0.8%
Chiu 2001	7	78	9.0	[2.6; 15.3]	0.5%
Chesney 2008	5	53	9.4	[1.6; 17.3]	0.3%
	341	.			0.0%
**Random effects model**		**25761**	**1.5**	**[1.1; 2.0]**	**100.0%**

Heterogeneity: $I^2 = 74\%$, $\tau^2 < 0.0001$, $\chi^2_{16} = 62.43$ ($p < 0.01$)

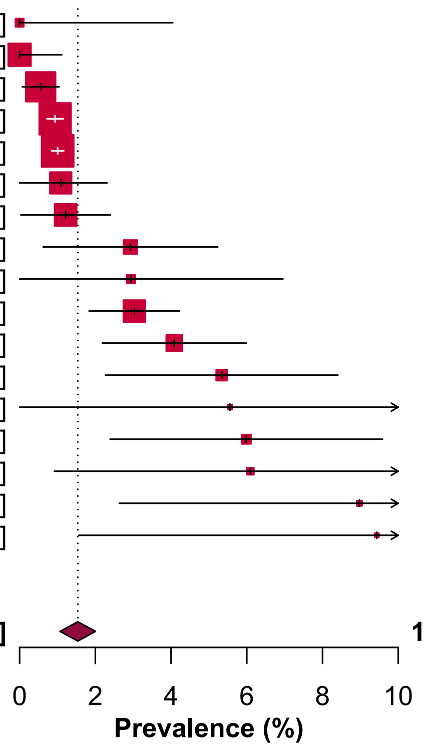

E

Study ID	Cases	Total	Prevalence	95% C.I.	Weight
**Diabetic: Excluding small studies**					
Teo 2018	0	123	0.0	[0.0; 1.1]	8.8%
Chan 2020	5	898	0.6	[0.1; 1.0]	16.5%
Adams 2013	71	7567	0.9	[0.7; 1.2]	19.7%
Namba 2013	145	14432	1.0	[0.8; 1.2]	20.1%
Suzuki 2010	3	276	1.1	[0.0; 2.3]	7.9%
Meding 2003	4	329	1.2	[0.0; 2.4]	8.2%
Iorio 2012	6	205	2.9	[0.6; 5.2]	3.1%
Song 2012	24	792	3.0	[1.8; 4.2]	8.2%
Iqbal 2019	17	416	4.1	[2.2; 6.0]	4.2%
Dowsey 2008	11	206	5.3	[2.3; 8.4]	1.9%
Han 2012	10	167	6.0	[2.4; 9.6]	1.4%
**Random effects model**		**25411**	**1.4**	**[0.9; 1.8]**	**100.0%**

Heterogeneity: $I^2 = 78\%$, $\tau^2 < 0.0001$, $\chi^2_{10} = 45.69$ ($p < 0.01$)

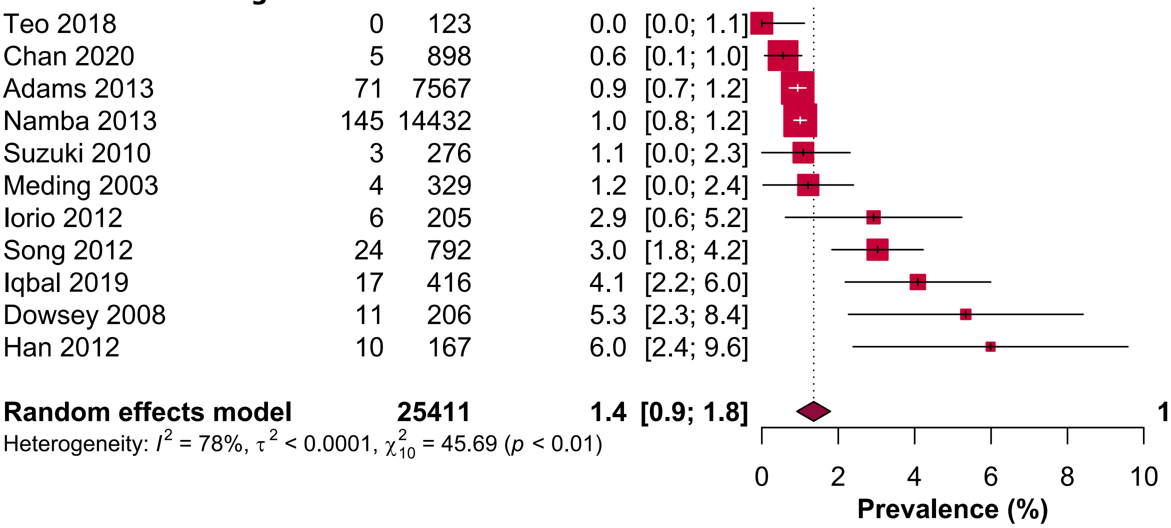

F

Study ID	Cases	Total	Prevalence	95% C.I.	Weight
**Diabetic: Excluding low- and moderate-quality studies**					
Teo 2018	0	123	0.0	[0.0; 1.1]	10.2%
Chan 2020	5	898	0.6	[0.1; 1.0]	13.4%
Adams 2013	71	7567	0.9	[0.7; 1.2]	14.3%
Namba 2013	145	14432	1.0	[0.8; 1.2]	14.4%
Suzuki 2010	3	276	1.1	[0.0; 2.3]	9.6%
Meding 2003	4	329	1.2	[0.0; 2.4]	9.8%
Papagelopoulos 1996	2	68	2.9	[0.0; 7.0]	2.2%
Song 2012	24	792	3.0	[1.8; 4.2]	9.7%
Iqbal 2019	17	416	4.1	[2.2; 6.0]	6.5%
Dowsey 2008	11	206	5.3	[2.3; 8.4]	3.4%
Font-Vizcarra 2011	2	36	5.6	[0.0; 13.0]	0.7%
Han 2012	10	167	6.0	[2.4; 9.6]	2.7%
Fan 2008	5	82	6.1	[0.9; 11.3]	1.4%
Chiu 2001	7	78	9.0	[2.6; 15.3]	1.0%
Chesney 2008	5	53	9.4	[1.6; 17.3]	0.6%
Blanco 2019	24	37	64.9	[49.5; 80.2]	0.2%
**Random effects model**		**25560**	**1.9**	**[1.3; 2.6]**	**100.0%**

Heterogeneity: $I^2 = 88\%$, $\tau^2 < 0.0001$, $\chi^2_{15} = 125.74$ ($p < 0.01$)

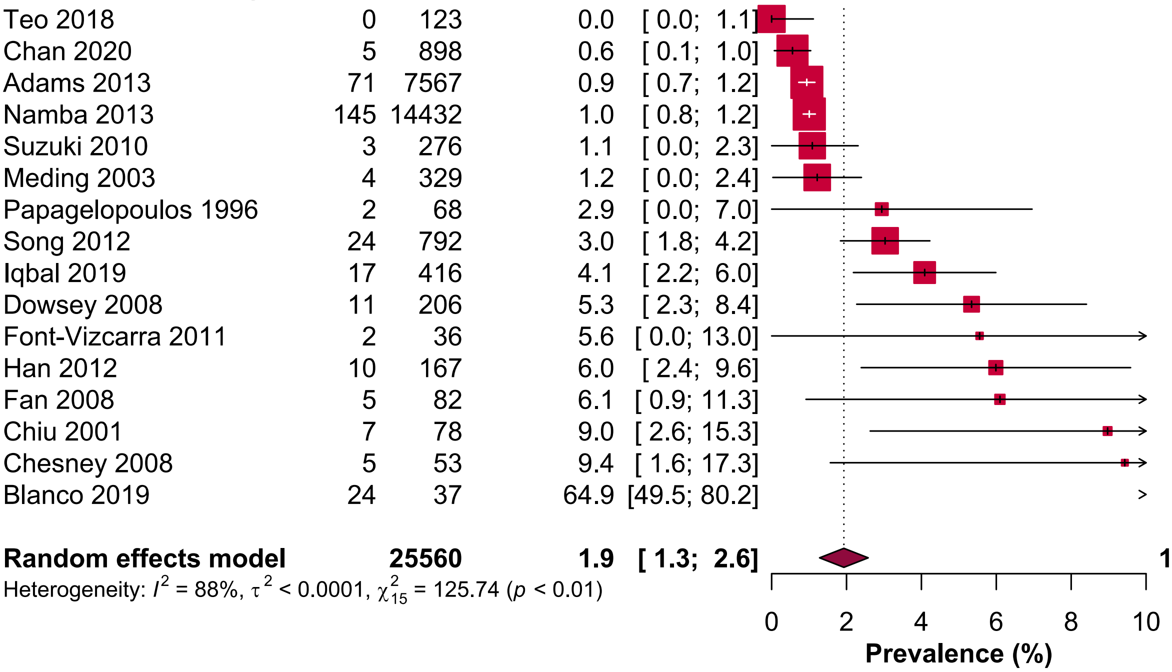

G

Study ID	Cases	Total	Prevalence	95% C.I.	Weight
**Non-diabetic: Excluding outlier studies**					
Namba 2013	259	41784	0.6	[0.5; 0.7]	13.1%
Adams 2013	216	32924	0.7	[0.6; 0.7]	13.0%
Dowsey 2008	7	1008	0.7	[0.2; 1.2]	7.9%
Chan 2020	8	1119	0.7	[0.2; 1.2]	8.1%
Meding 2003	35	4891	0.7	[0.5; 1.0]	11.6%
Suzuki 2010	14	1746	0.8	[0.4; 1.2]	9.1%
Iqbal 2019	34	3853	0.9	[0.6; 1.2]	10.8%
Iorio 2012	26	2274	1.1	[0.7; 1.6]	8.8%
Teo 2018	10	782	1.3	[0.5; 2.1]	5.1%
Song 2012	59	2634	2.2	[1.7; 2.8]	7.2%
Fan 2008	9	390	2.3	[0.8; 3.8]	1.9%
Chesney 2008	60	1279	4.7	[3.5; 5.8]	2.9%
Font-Vizcarra 2011	9	177	5.1	[1.8; 8.3]	0.5%
**Random effects model**		**94861**	**1.1**	**[0.8; 1.3]**	**100.0%**

Heterogeneity: $I^2 = 88\%$, $\tau^2 < 0.0001$, $\chi^2_{12} = 98.45$ ($p < 0.01$)

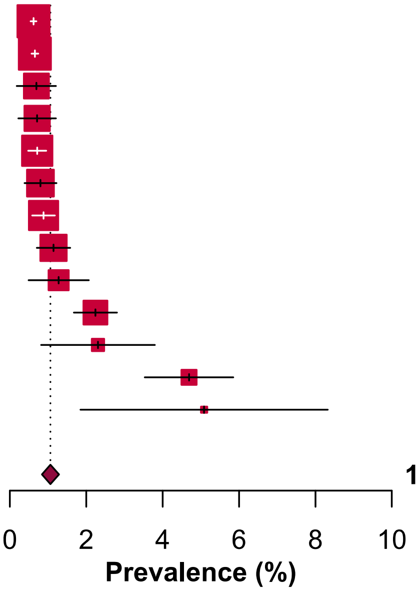

H

Study ID	Cases	Total	Prevalence	95% C.I.	Weight
**Non-diabetic: Excluding small studies**					
Namba 2013	259	41784	0.6	[0.5; 0.7]	11.4%
Adams 2013	216	32924	0.7	[0.6; 0.7]	11.4%
Dowsey 2008	7	1008	0.7	[0.2; 1.2]	8.3%
Chan 2020	8	1119	0.7	[0.2; 1.2]	8.5%
Meding 2003	35	4891	0.7	[0.5; 1.0]	10.6%
Suzuki 2010	14	1746	0.8	[0.4; 1.2]	9.2%
Iqbal 2019	34	3853	0.9	[0.6; 1.2]	10.2%
Iorio 2012	26	2274	1.1	[0.7; 1.6]	9.0%
Teo 2018	10	782	1.3	[0.5; 2.1]	6.1%
Song 2012	59	2634	2.2	[1.7; 2.8]	7.9%
Fan 2008	9	390	2.3	[0.8; 3.8]	2.7%
Chesney 2008	60	1279	4.7	[3.5; 5.8]	3.9%
Font-Vizcarra 2011	9	177	5.1	[1.8; 8.3]	0.7%
Blanco 2019	42	95	44.2	[34.2; 54.2]	0.1%
**Random effects model**		**94956**	**1.2**	**[0.9; 1.5]**	**100.0%**

Heterogeneity: $I^2 = 92\%$, $\tau^2 < 0.0001$, $\chi^2_{13} = 171.41$ ($p < 0.01$)

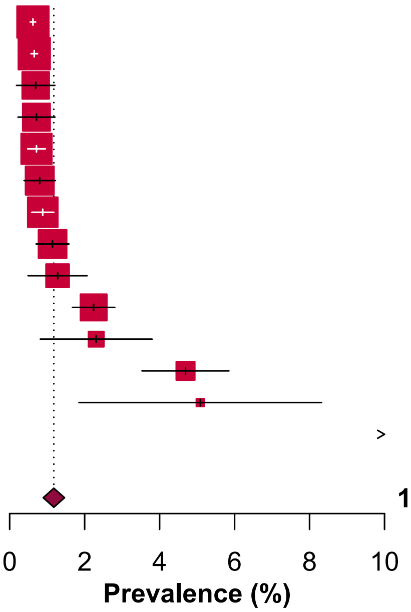

I

Study ID	Cases	Total	Prevalence	95% C.I.	Weight
**Non-diabetic: Excluding low- and moderate-quality studies**					
Namba 2013	259	41784	0.6	[0.5; 0.7]	12.5%
Adams 2013	216	32924	0.7	[0.6; 0.7]	12.4%
Dowsey 2008	7	1008	0.7	[0.2; 1.2]	9.2%
Chan 2020	8	1119	0.7	[0.2; 1.2]	9.4%
Meding 2003	35	4891	0.7	[0.5; 1.0]	11.7%
Suzuki 2010	14	1746	0.8	[0.4; 1.2]	10.1%
Iqbal 2019	34	3853	0.9	[0.6; 1.2]	11.2%
Teo 2018	10	782	1.3	[0.5; 2.1]	6.7%
Song 2012	59	2634	2.2	[1.7; 2.8]	8.7%
Fan 2008	9	390	2.3	[0.8; 3.8]	3.0%
Chesney 2008	60	1279	4.7	[3.5; 5.8]	4.3%
Font-Vizcarra 2011	9	177	5.1	[1.8; 8.3]	0.8%
Blanco 2019	42	95	44.2	[34.2; 54.2]	0.1%
**Random effects model**		**92682**	**1.2**	**[0.9; 1.5]**	**100.0%**

Heterogeneity: $I^2 = 93\%$, $\tau^2 < 0.0001$, $\chi^2_{12} = 167.18$ ($p < 0.01$)

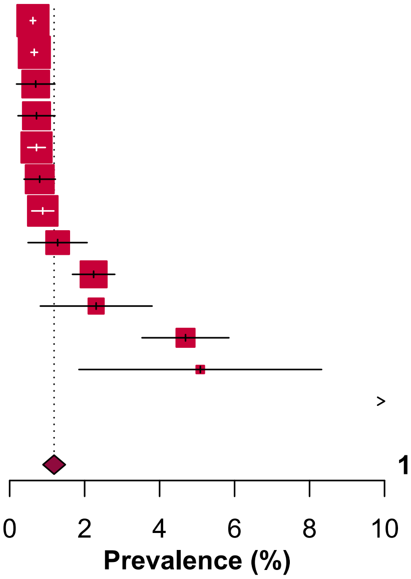

Figure S1. Sensitivity analyses.

Table S1. Search strategies

Databases	Search strategies
PubMed	(knee replacement[Title/Abstract] OR knee replacements[Title/Abstract] OR knee arthroplasty[Title/Abstract] OR knee arthroplasties[Title/Abstract] OR TKA[Title/Abstract] OR TKR[Title/Abstract]) AND (Diabetic[Title/Abstract] OR Diabetics[Title/Abstract] OR diabetes[Title/Abstract])
Scopus	TITLE-ABS("knee replacement" OR "knee replacements" OR "knee arthroplasty" OR "knee arthroplasties" OR TKA OR TKR) AND TITLE-ABS(Diabetic OR Diabetics OR diabetes)
Web of Science	TI=("knee replacement" OR "knee replacements" OR "knee arth roplasty" OR "knee arthroplasties" OR TKA OR TKR) AND TI=(Diabetic OR Diabetics OR diabetes)<br>*Indexes=SCI-EXPANDED, SSCI, A&HCI, CPCI-S, CPCI-SSH, BKCI-S, BKCI-SSH, ESCI Timespan=All years*
ScienceDirect	Title, abstract, keywords: ("knee replacement" OR "knee replacements" OR "knee arthroplasty" OR "knee arthroplasties" OR TKA OR TKR) AND (Diabetic OR Diabetics OR diabetes)
Google Scholar	allintitle:("knee replacement" OR "knee replacements" OR "knee arthroplasty" OR "knee arthroplasties" OR TKA OR TKR) (Diabetic OR Diabetics OR diabetes)

Table S2. Quality assessment of the included case-control studies

No.	Study ID	Questions assessing the included case-control studies										Yes (%)
		1	2	3	4	5	6	7	8	9	10	
1	Adams 2013	Y	U	Y	Y	Y	Y	Y	Y	Y	Y	90.0
2	Blancho 2019	Y	Y	Y	Y	Y	Y	U	Y	U	Y	80.0
3	Chan 2020	Y	Y	Y	Y	Y	U	Y	U	Y	Y	80.0
4	Chesney 2008	Y	U	Y	Y	Y	Y	Y	Y	Y	Y	90.0
5	Dowsey 2008	Y	U	Y	Y	Y	Y	Y	Y	Y	Y	90.0
6	Fan 2008	Y	U	Y	Y	Y	Y	U	Y	Y	Y	80.0
7	Font-Vizcarra 2011	Y	U	Y	U	Y	Y	Y	Y	Y	Y	80.0
8	Iorio 2012	Y	U	Y	Y	Y	U	U	U	Y	Y	60.0
9	Iqbal 2019	Y	U	Y	U	Y	Y	Y	Y	Y	Y	80.0
10	Meding 2003	Y	U	Y	Y	Y	Y	Y	U	Y	Y	80.0
11	Namba 2013	Y	U	U	Y	Y	Y	Y	Y	Y	Y	80.0
12	Song 2012	Y	U	Y	U	Y	Y	Y	Y	Y	Y	80.0
13	Suzuki 2010	Y	U	Y	Y	Y	Y	Y	U	Y	Y	80.0
14	Teo 2018	Y	U	Y	Y	Y	Y	Y	U	Y	Y	80.0

1. Were the groups comparable other than the presence of disease in cases or the absence of disease in controls? 2. Were cases and controls matched appropriately? 3. Were the same criteria used for identification of cases and controls? 4. Was exposure measured in a standard, valid and reliable way? 5. Was exposure measured in the same way for cases and controls? 6. Were confounding factors identified? 7. Were strategies to deal with confounding factors stated? 8. Were outcomes assessed in a standard, valid and reliable way for cases and controls? 9. Was the exposure period of interest long enough to be meaningful? 10. Was appropriate statistical analysis used? Y=Yes; N=No; U=Unclear.

Table S3. Quality assessment of the included cohort studies

No.	Study ID	Questions assessing the included cohort studies											Yes (%)
		1	2	3	4	5	6	7	8	9	10	11	
1	Chiu 2001	Y	Y	Y	Y	Y	Y	Y	Y	Y	U	Y	90.9
2	Han 2012	Y	Y	Y	Y	Y	Y	Y	Y	U	U	Y	81.8
3	Papagelopoulos 1996	Y	U	U	Y	Y	Y	U	Y	Y	Y	Y	72.7
4	Ravindran 2020	Y	Y	Y	Y	U	Y	U	Y	U	U	Y	63.6

1. Were the two groups similar and recruited from the same population? 2. Were the exposures measured similarly to assign people to both exposed and unexposed groups? 3. Was the exposure measured in a valid and reliable way? 4. Were confounding factors identified? 5. Were strategies to deal with confounding factors stated? 6. Were the groups/participants free of the outcome at the start of the study (or at the moment of exposure)? 7. Were the outcomes measured in a valid and reliable way? 8. Was the follow up time reported and sufficient to be long enough for outcomes to occur? 9. Was follow up complete, and if not, were the reasons to loss to follow up described and explored? 10. Were strategies to address incomplete follow up utilized? 11. Was appropriate statistical analysis used? Y=Yes; N=No; U=Unclear, NA=Not applicable.
